# Tissue‐Penetrating Ultrasound‐Triggered Hydrogel for Promoting Microvascular Network Reconstruction

**DOI:** 10.1002/advs.202401368

**Published:** 2024-04-10

**Authors:** Zhenyu Zhao, Yin Zhang, Chen Meng, Xiaoyun Xie, Wenguo Cui, Keqiang Zuo

**Affiliations:** ^1^ Department of Interventional and Vascular Surgery Shanghai Tenth People's Hospital Tongji University School of Medicine Shanghai 200072 China; ^2^ Department of Orthopaedics Shanghai Key Laboratory for Prevention and Treatment of Bone and Joint Diseases Shanghai Institute of Traumatology and Orthopaedics Ruijin Hospital Shanghai Jiao Tong University School of Medicine 197 Ruijin 2nd Road Shanghai 200025 China

**Keywords:** gene therapy, injectable hydrogel, microvascular network, tissue penetration, ultrasound

## Abstract

The microvascular network plays an important role in providing nutrients to the injured tissue and exchanging various metabolites. However, how to achieve efficient penetration of the injured tissue is an important bottleneck restricting the reconstruction of microvascular network. Herein, the hydrogel precursor solution can efficiently penetrate the damaged tissue area, and ultrasound triggers the release of thrombin from liposomes in the solution to hydrolyze fibrinogen, forming a fibrin solid hydrogel network in situ with calcium ions and transglutaminase as catalysts, effectively solving the penetration impedance bottleneck of damaged tissues and ultimately significantly promoting the formation of microvascular networks within tissues. First, the fibrinogen complex solution is effectively permeated into the injured tissue. Second, ultrasound triggered the release of calcium ions and thrombin, activates transglutaminase, and hydrolyzes fibrinogen. Third, fibrin monomers are catalyzed to form fibrin hydrogels in situ in the damaged tissue area. In vitro studies have shown that the fibrinogen complex solution effectively penetrated the artificial bone tissue within 15 s after ultrasonic triggering, and formed a hydrogel after continuous triggering for 30 s. Overall, this innovative strategy effectively solved the problem of penetration resistance of ultrasound‐triggered hydrogels in the injured tissues, and finally activates in situ microvascular networks regeneration.

## Graphical abstract

In the study, ultrasound triggers the release of thrombin from liposomes in the solution to hydrolyze fibrinogen, forming a fibrin solid hydrogel network in situ with calcium ions and transglutaminase as catalysts, effectively solving the penetration impedance bottleneck of damaged tissues and ultimately significantly promoting the formation of microvascular networks within tissues.

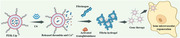

## 1 Introduction

The microvascular network, a complex circulatory system, efficiently transports gases, nutrients, and metabolites to and from cells.^[^ ^1^ ^]^ This system is pivotal in injured tissues, where incomplete Vascular Endothelial Growth Factor (VEGF) signaling may drive organ tissue aging and damage.^[^ ^2^ ^]^ Conversely, elevating VEGF signaling can prevent age‐related capillary loss, improving perfusion and function in various injured tissues, ultimately extending lifespan.^[^ ^3^ ^]^ In addition, Fu et al.^[^ ^4^ ^]^ found that Zinc Finger E‐box‐binding Homeobox Protein 1 (ZEB1) deletion in injured tissue reduced histone acetylation on the promoter, thereby inhibiting the angiogenesis signaling pathway and leading to a reduction in the formation of microvessel in injured tissue. This study further reveals the close relationship between microvascular network generation and the development of injured tissues. Studies indicate that osteoporosis results in significant bone damage, including destruction, altered microstructure, and reduced microvascular network activity.^[^ ^5^ ^]^ Microvascular network is closely related to the imbalance of bone cell metabolism and abnormal bone development in injured tissues.^[^ ^6^ ^]^ Furthermore, microvascular network reconstruction is essential for repairing various injured tissues, ensuring adequate blood supply during regeneration and expansion.^[^ ^7^ ^]^ Although the current study highlights its importance, challenges remain in effectively achieving microvascular network reconstruction.

Therefore, to effectively solve the technical problem of microvascular network reconstruction in damaged tissues, in‐depth research has been carried out at home and abroad.^[^ ^8^ ^]^ The regeneration of the microvascular network has great potential in the regeneration and clinical treatment of damaged tissues.^[^ ^9^ ^]^ Therefore, in clinical treatment, tissue transplantation with vascular pedicle is often used to repair damaged tissues.^[^ ^10^ ^]^ Healthy microvascularized tissue transplantation can allow capillaries to penetrate damaged tissues, resulting in a higher success rate of in situ repair.^[^ ^11^ ^]^ However, some donor tissues that are challenging to transplant, or feature underdeveloped microvascular networks and poor tissue permeability after post‐transplantation. These factors are making the clinical technique greatly limited.^[^ ^12^ ^]^ In addition, there are many molecular biology studies devoted to the reconstruction of microvascular network in injured tissues. The reconstruction of microvascular network system must involve key endothelial cells.^[^ ^13^ ^]^ Studies have found that angiogenic factors such as vascular endothelial growth factor, platelet‐derived growth factor‐AA, and platelet‐derived growth factor‐BB can activate endothelial cells and further promote vascular reconstruction.^[^ ^14^ ^]^ However, the activity of these vasoactive factors is difficult to maintain, and it is even more difficult to effectively penetrate the injured tissue and continuously promote the in situ reconstruction of the microvascular network.^[^ ^15^ ^]^ In addition, mechanism studies reveal that gene overexpression of ZEB1 can activate ZEB1/Notch signaling pathway to promote vascular regeneration.^[^ ^4^ ^]^ However, gene therapy requires frequent systemic injections, which making it difficult to slow release and effectively penetrate local injured tissues to achieve microvascular network reconstruction. Therefore, the existing clinical microvascular network reconstruction of injured tissue still has the problem of poor tissue permeability, and it is difficult to make an effective breakthrough.

In recent years, tissue engineering technology has been applied in the field of microvascular network reconstruction.^[^ ^16^ ^]^ The principal objective is to construct a regenerative microvascular network capable of penetrating well within injured tissues, facilitating in situ regeneration and matching well with natural blood vessels well.^[^ ^17^ ^]^ Over the past few years, the biomaterials has evolved to mimic the extracellular matrix of blood vessels,^[^ ^18^ ^]^ because the interaction between vascular cells and the extracellular matrix could recapitulate the structure and function of vascular network.^[^ ^19^ ^]^ Although the biomaterials has a strong effect on the regeneration of large blood vessels, there are still many challenges in tissue penetration and reconstruction of vascular network at microscopic level.^[^ ^20^ ^]^ In addition, 3D‐printed scaffolds or hydrogel combining with blood vessel cells, growth factors, and gene drugs provides the appropriate biological, physical, and structural clues for blood vessel growth to ensure release slowly and promote microvascular reconstruction steadily.^[^ ^21^ ^]^ However, this method also has limitations that the tissue permeability of biological materials is poor and can only be implanted in the injured tissue area. Recently, the integration of ultrasound technology and functional biomaterials has shown promise in achieving precise treatment in terms of dose, space, and time.^[^ ^22^ ^]^ The use of ultrasound technology could be facilitating efficient penetration of liquids in tissues and triggering the in situ formation of solid‐state hydrogels, which will activate drug delivery.^[^ ^23^ ^]^ However, current research has a poor ability to utilize ultrasound to trigger the formation of biomaterials within tissues and promote the regeneration of microvascular networks in situ, making it difficult to achieve the reconstruction of microvascular networks in damaged tissues.

**Figure 1:**
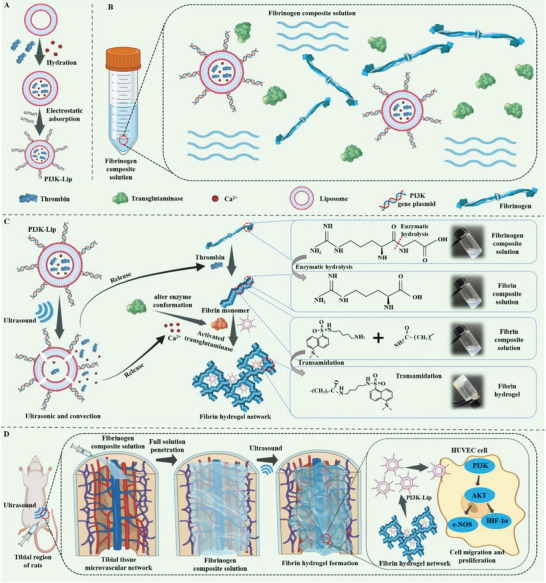
Schematic Figure of ultrasound‐triggered tissue permeable hydrogels. A) Preparation process of PI3K liposomes. B) Composition of fibrinogen complex solution. C) Ultrasound triggers the release of thrombin from liposomes in the solution to hydrolyze fibrinogen, forming a fibrin solid hydrogel network in situ with calcium ions and transglutaminase as catalysts. D) Tissue infiltration ultrasound‐triggered hydrogel in vivo formation process and mechanism of promoting microvessel formation.

Local tissue genetic engineering techniques provide a powerful means for exploring gene function and the potential of microvascular network regeneration.^[^ ^24^ ^]^ However, the absence of effective triggering techniques and advanced vectors impede the successful reconstruction of microvascular networks within tissues.^[^ ^25^ ^]^ Herein, the hydrogel precursor solution can penetrate the damaged tissue area efficiently, and after ultrasound triggering, liposomes release thrombin to hydrolyze fibrinogen. With calcium ions and transglutaminase as catalysts, a solid fibrin hydrogel network is triggered to form in situ, solving the penetration impedance bottleneck of damaged tissues and significantly promoting the formation of microvascular networks within the tissue. First, a mixture of fibrinogen solution was injected into the injured tissue area so that it fully penetrates the injured tissue and its surrounding microvascular network (**Figure** **1**A,B). Subsequently, ultrasound triggered liposomes to release calcium ions and thrombin. Calcium ion changed the energy coordination of transglutaminase, leading to its conformational change, and its conformational change could open the channel and make it activate. Thrombin catalyzed the hydrolysis of fibrinogen to fibrin monomers (Figure 1C). The fibrin monomer was catalytically crosslinked by activated transglutaminase through transamidation reactions, resulting in covalent crosslinking of the lysine and glutamine side chain residues of the fibrin molecules, and simultaneously binding with liposomes carrying the Glyceraldehyde‐3‐Phosphate Dehydrogenase (PI3K) gene, forming a fibrin network in situ in the damaged tissue area (Figure 1C). Eventually, an ultrasound‐triggered in situ crosslinked hydrogel permeating damaged tissues was constructed, which promoted the reconstruction of the microvascular network in the damaged tissue area by locally activating the PI3K‐AKT pathway through genetic engineering (Figure 1D). In vitro studies, the hydrogel precursor solution was added to the artificial bone, and the ultrasound continuously triggered the effective penetration of the bone tissue within 15 s, and the rapid formation of the hydrogel within 30 s. More remarkably, the hydrogel also showed good in vitro promotion of vascularization function. In vivo studies revealed that the system could effectively penetrate the tibia and continuously promote the reconstruction of the microvascular network within the tibia for 8 weeks. Therefore, the advanced tissue‐permeable ultrasound‐triggered hydrogel we constructed could effectively solve the treatment bottleneck of penetration impedance of injured tissues and has great therapeutic potential in the reconstruction of microvascular networks.

## 2 Results

### 2.1 Liposome Preparation and Transfection Efficiency Evaluation

**Figure 2:**
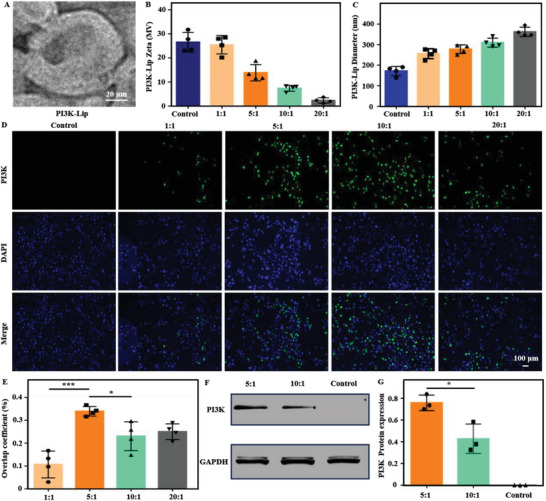
Characterization of complex liposomes and evaluation of transfection effect. A) SEM images of liposomes. Scale bar: 20 µm. B,C) Size and zeta potential evaluation of liposomes. D,E) Fluorescence expression of liposome complexes with different N/P after transfection, and related transfection efficiency evaluation (blue: DAPI staining of nuclei; Green: green fluorescence expression of target gene). Scale bar: 100 µm. F,G) Protein immunoimprinting was used to evaluate the expression of PI3K gene after transfection.Data are reported as mean ± SD, ^*^ *p* < 0.05, ^***^ *p* < 0.001.

The hydrogel formation process triggered by the ultrasound and the mechanism promoting angiogenesis were shown in Figure 1. We prepared PI3K‐LIP liposomes by electrostatic adsorption, which loaded Ca^2+^, thrombin and PI3K gene. Transmission electron microscopy (TEM) indicated that the liposome showed a good phospholipid bilayer structure (**Figure** **2**A). Dynamic light scattering (DLS) was used to investigate the particle size and Zeta potential change of the liposomes. The results showed that the surface charge and size of the liposomes increased with the increasing proportion of complementary DNA (cDNA) (Figure 2B,C). The transfection efficiency of liposome complexes with various cDNA/Liposome (N/P) ratios was assessed using fluorescence microscopy. After the liposome complex was co‐cultured with cells for 48 h, the overlapping efficiency of nuclear blue fluorescence and green fluorescence was quantitatively detected using Image J (Figure 2D,E). The findings demonstrated that the fluorescence overlap coefficient and transfection efficiency were greatest when the N/P ratio was 5:1. In addition, Western blot results showed that PI3K protein was successfully expressed after transfection of Human umbilical cord blood endothelial cells (HUVECs) with liposome complex (Figure 2F,G), and the expression of N/P protein at 5:1 was higher than that at 10:1. Besides, the N/P ratio of 5:1 was proved to be a more suitable liposomal transfection ratio. Thus, these results indicated that we have successfully developed functional complex liposomes capable of efficiently transfecting the PI3K gene into HUVECs.

### 2.2 Preparation and Characterization of Ultrasound‐Triggered Tissue Permeable Hydrogels

**Figure 3:**
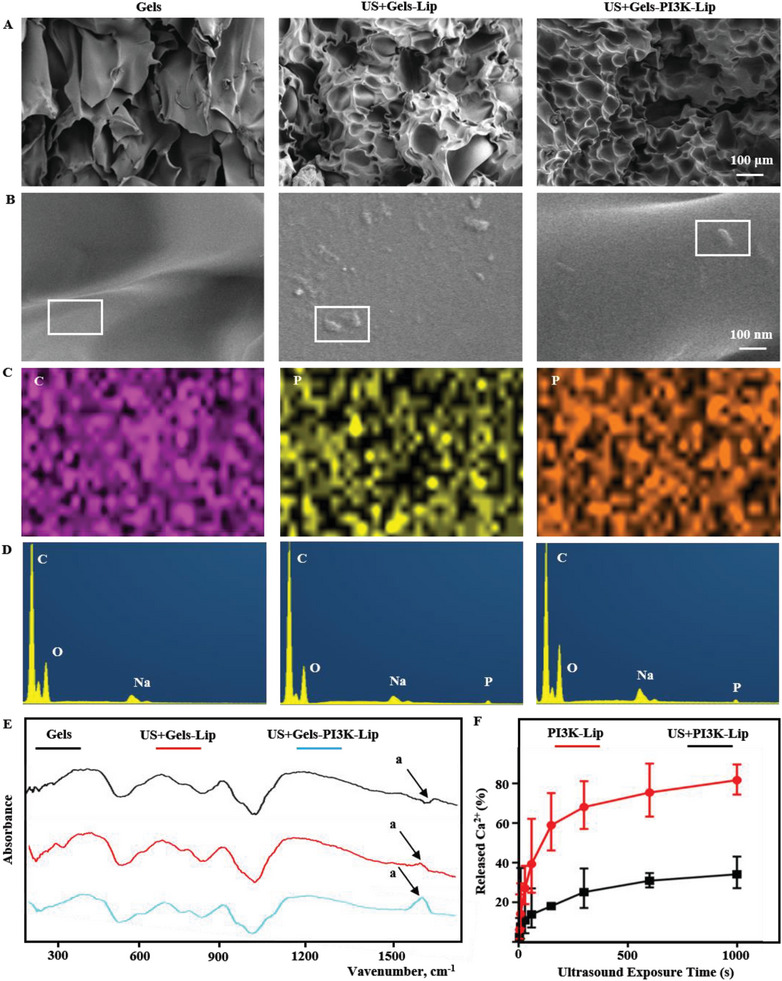
Related characterization of tissue‐penetrating ultrasound‐triggered hydrogel. A,B) SEM was used to detect the surface morphology of hydrogel. Scale bar: 100 µm and Scale bar: 100 nm. C,D) The distribution of related elements was detected in EDS mode. The grouping is consistent with parts A and B. E) FTIR detects changes in relevant chemical groups. F) The release of calcium ions after ultrasonic triggering.

The surface morphology, elemental composition, chemical bond alterations, and calcium ion release efficiency were crucial aspects of hydrogel. SEM showed that the surface of the hydrogel was porous and liposome structures could be found on the surface of the hydrogel (**Figure** **3**A,B). Furthermore, Energy dispersive spectroscopy (EDS) result demonstrated the presence of P elements in the Ultrasound+Gelatin+Liposome (US+Gels‐LIP) and US+Gels‐PI3K‐LIP groups. There was no liposome structure or P element distribution on the surface of the gel group (Figure 3C,D). These results indicated that the liposomes were successfully loaded on the surface of hydrogel. The hydrogen‐bond interaction between liposomes and fibrin monomers can promote the formation of hydrogels. Fourier Transform Infrared (FTIR) results showed that the hydrogen bond peak of US+Gels‐LIP group and US+Gels‐PI3K‐LIP group was significantly changed at 1596 cm‐1 compared with the Gels group without liposomes a). Therefore, it was further proved that the US+Gels‐Lip and US+Gels‐PI3K‐Lip groups successfully loaded liposomes (Figure 3E).^[^ ^26^ ^]^ In addition, Figure 3F showed that US+PI3K‐Lip can release calcium ions rapidly within 50 s after ultrasonic triggering, thus activating transglutaminase formation and triggering cross‐linking between fibrin monomers. Therefore, we also found the rapid formation of hydrogels within 30 s of ultrasound (Figure S1, Supporting Information).

### 2.3 Mechanical Properties Characterization of Ultrasound‐Triggered Tissue Permeability Hydrogels

**Figure 4:**
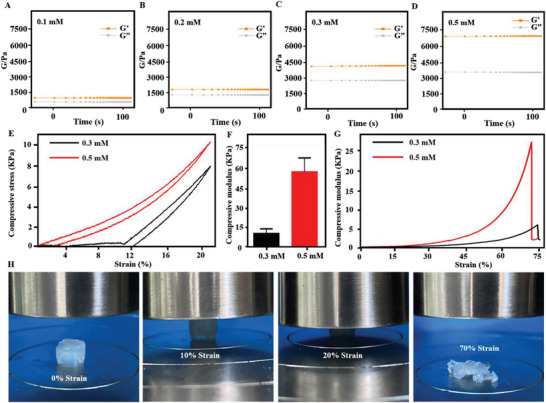
Characterization of mechanical properties of tissue‐penetrating ultrasound‐triggered hydrogel. A–D) Hydrogel modulus contained in different solids. E,F) Stress–strain curves of hydrogels. G) Fracture tests of hydrogels. H) Status of hydrogels in different compression modes.

We systematically investigated the mechanical properties of hydrogels in different groups. **Figure** **4**A–D showed the energy storage modulus (G′) and loss modulus (GG″) of hydrogel. We found that when the content of hydrogel solids increases, the modulus of hydrogel also increases. Subsequently, we investigated the compressive stress–strain curves and mechanical properties of 0.3 and 0.5 mm hydrogels. Subsequently, we investigated the compressive stress–strain curves and mechanical properties of 0.3 and 0.5 mm hydrogels (Figure 4E). Quantitative statistical analyses of the compression and modulus of hydrogels found that hydrogel fracture occurred at 70% compression rate (Figure 4F–H). The relevant compression modulus was also quantitatively analyzed.

### 2.4 Biocompatibility Evaluation of Ultrasound‐Triggered Tissue Permeability Hydrogels

**Figure 5:**
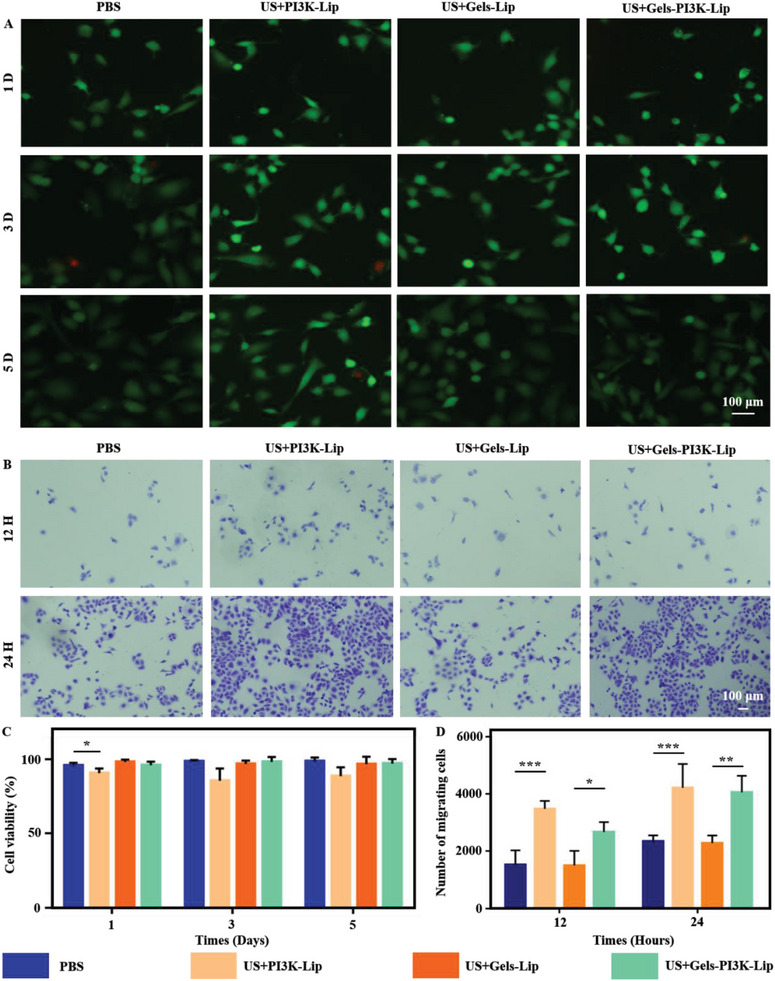
Evaluation of biocompatibility of tissue‐penetrating ultrasound‐triggered hydrogel. A,C) Biocompatibility of hydrogels was assessed using live/dead staining and relevant statistical analyses were performed (Red: Dead cells; Green: Living cells). Scale bar: 100 µm. B,D) Evaluation of cell migration efficiency in Transwell plates. Scale bar: 100 µm. Data are reported as mean ± SD, ^*^ *p* < 0.05, ^**^ *p* < 0.01, ^***^ *p* < 0.001.

Biocompatibility was a crucial in biomaterials assessment.^[^ ^27^ ^]^ The effects of the hydrogel on the bioactivity of endothelial cells were evaluated through live/dead tests and relevant statistical analyses. **Figure** **5**A,C revealed that the US+PI3K‐Lip group possessed greater biotoxicity than other groups due to the lack of sustained‐release effect (P < 0.05). Notably, the image of the nucleus and skeleton of the stained endothelial cells co‐cultured with the hydrogel suggested that hydrogels exhibit good biocompatibility and can be used for further study (Figure S2, Supporting Information).

### 2.5 Exploration of Cell Migration In Vitro

**Figure 6:**
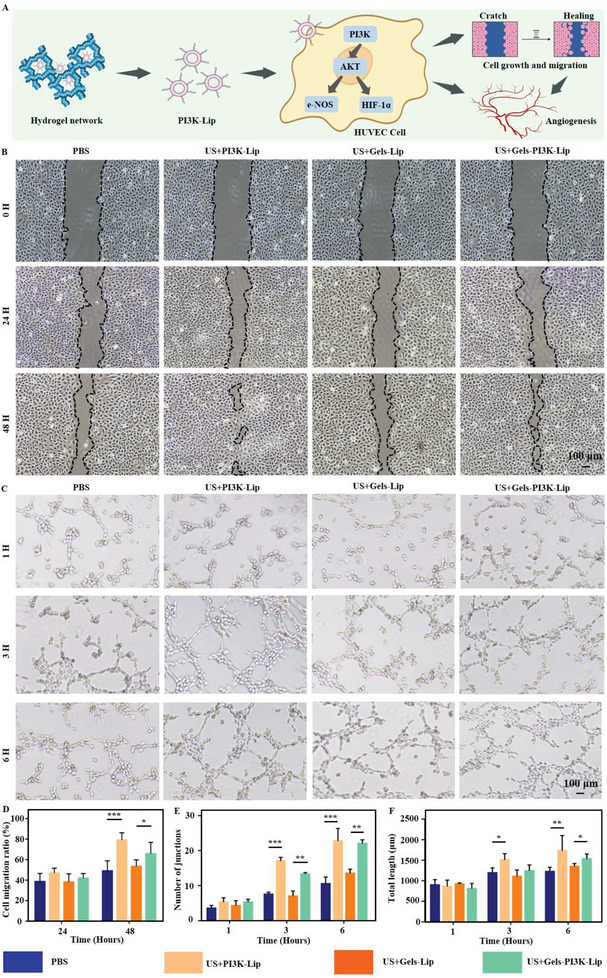
The migration efficiency and angiogenic properties of hydrogels were evaluated. A) Functional evaluation of biomaterials promoting cell migration and angiogenesis. B,D) Scratch tests were performed to evaluate cell migration performance and relevant statistical analyses were performed. Scale bar: 100 µm. C) Angiogenesis experiment was performed to evaluate angiogenesis performance in vitro. Scale bar: 100 µm. E,F) and relevant statistical analyses were performed. Data are reported as mean ± SD, ^*^ *p* < 0.05, ^**^ *p* < 0.01, ^***^ *p* < 0.001.

US+Gels‐PI3K‐Lip can activate the PI3K/AKT signaling pathway, influencing cell migration. The migration of endothelial cells was investigated to determine the effect of materials in each group on cell migration via scratch test (Figure 6A). Compared to Phosphate Buffer Saline (PBS) and US+Gels‐Lip, the US+Gels‐PI3K‐Lip group exhibited significantly increased cell mobility (P < 0.05), as depicted in Figure 5B,D. Additionally, cell migration was more pronounced in the US+PI3K‐Lip group due to the lack of slow‐release effect of hydrogel. The hydrogel's lack of a slow‐release effect led to the transfer of numerous liposomes into cells, resulting in a relatively significant short‐term in vitro treatment. Transwell experiment was conducted to investigated the migration of endothelial cells in each group. The results showed that there were no significant differences in cell migration levels among all groups at 24 h. The migration levels of US+PI3K‐Lip and US+Gels‐PI3K‐Lip were significantly increased compared with other culture groups at the 48‐h time node (P <0.05) (Figure 6B,D). Therefore, the above results suggested that the stimulation of the complex hydrogel can effectively promote the migration of HUVECs.

### 2.6 Evaluation of Ultrasound‐Triggered Tissue‐Permeable Hydrogels for Promoting In Vitro Vascularization

To verify the in vitro angiogenesis of US+Gel‐PI3K‐Lip, HUVECs were co‐cultured with extracts of materials from each group, and the in vitro angiogenesis performance was shown in Figure 6C. In addition, relevant statistical analysis evaluation was conducted (Figure 6E,F). The results showed that, compared with PBS group, co‐culture of cells with US+Gel‐PI3K‐Lip group could effectively promote angiogenesis. This can be demonstrated by an increase in the number of cell connections and overall length. In addition, the in vitro angiogenesis performance of US+Gel‐PI3K‐Lip group was significantly higher. These results indicated that US+Gel‐PI3K‐Lip hydrogel had excellent angiogenic properties in vitro (Figure S3, Supporting Information).

### 2.7 Evaluation of Tissue‐Permeable Hydrogels for Promoting In Vivo Microvascular Reconstruction

**Figure 7:**
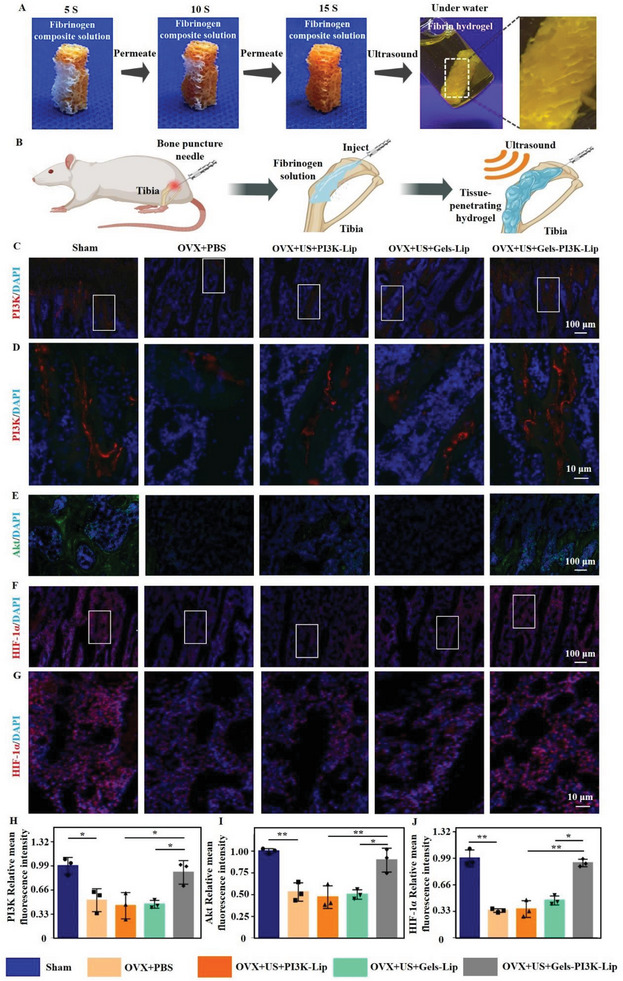
Evaluation of ultrasound‐triggered tissue permeable hydrogels promoting microvascular remodeling in vivo. A) Permeability of fibrinogen complex solution in artificial bone and in liquid after crosslinking. B) Schematic of animal experiments. C,D). PI3K immunofluorescence expression, H) and quantitative analysis (red: PI3K gene fluorescence staining) Scale bar: 100 µm and Scale bar: 10 µm. E) Immunofluorescence expression of Akt, and I) quantitative analysis (green: Akt gene fluorescence staining). Scale bar: 100 µm. F,G) HIF‐1*α* immunofluorescence expression, Scale bar: 100 µm and Scale bar: 10 µm, J) and quantitative analysis (red: HIF‐1*α* gene fluorescence staining). Data are reported as mean ± SD, ^*^ *p* < 0.05, ^**^ *p* < 0.01.

The above in vitro studies confirmed that the hydrogel had a good PI3K gene transfection and treatment effect. Furthermore, we investigated its potential to activate the PI3K‐AKT signaling pathway in vivo, promoting HUVEC cell proliferation and migration. The solution could rapidly penetrate the artificial bone within 15 s when the solution was dripped onto the artificial bone. Upon ultrasonic triggering, hydrogel was efficiently formed in the artificial bone and remained stable in the liquid environment (Figure 7A). Over the past several years, numerous studies have accomplished the goal of local treatment through minimally invasive injection of biomaterials into the tibia.^[^ ^28^ ^]^ In addition, domestic and foreign clinical therapeutic instruments including ArrowEZ‐IO and Shusuda have been used for local injection of diverse mixed solutions (Figure 7B). Transfection effect of hydrogels in vivo was assessed via PI3K immunofluorescence staining and quantitative analysis were conducted initially (Figure 7C,D,H). The expression of AKT protein was analyzed by immunofluorescence (Figure 7E,I). Hypoxia‐Inducible Factor‐1*α* (HIF‐1*α*), an important protein in the PI3K‐AKT signaling pathway was investigated, which promoted angiogenesis (Figure 7F,G,J). Besides, the PI3K‐AKT signaling pathway affecting cell migration through Endothelial nitric oxide synthase (e‐NOS) protein signaling were also explored (Figure S4, Supporting Information). Our research demonstrated that the hydrogel modulates migration and proliferation of endothelial cells through the PI3K‐AKT signaling pathway.

## 3 Discussion

In the study, our hydrogel precursor solution efficiently penetrated the damaged tissue area, and subsequently ultrasound triggered the release of thrombin from liposomes in the solution to hydrolyze fibrinogen, and triggered the formation of fibrin solid hydrogel network in situ with calcium ions as well as transglutaminase as catalysts. In addition, many studies at home and abroad also use hydrogels to explore bone microvascular reconstruction. Zeng et al.^[^ ^29^ ^]^ developed an injectable gelatin hydrogel microsphere‐hydrogel complex as a stable drug delivery system for the treatment of critical femoral defects in rats. Sustained release of ferriamine in severe femoral defects stimulated the formation of functional H‐type vessels. This study provided a new theoretical basis for the treatment of large femoral defect by targeting H‐type vessels. In addition, Zhang et al.^[^ ^30^ ^]^ developed a covalently crosslinked amination bioactive glass composite hydrogel with angiogenic properties, which significantly improved the mechanical properties of the composite hydrogel. This study provided new evidence for the promotion of bone vascular regeneration by covalent crosslinked composite hydrogels with strong mechanical properties. Qiu et al.^[^ ^31^ ^]^ manufactured an injectable periosteal extracellular matrix hydrogel that promotes bone repair by enhancing blood vessels and osteogenesis through early immune regulation. Therefore, the hydrogel can promote bone vascular regeneration through immune regulation. Li et al.^[^ ^32^ ^]^ developed a PH‐responsive putaminal structure micro/nano hydrogel microsphere. The system achieved oral administration, gastric protection, intestinal sustained/controlled release, and H‐type vascular therapy in bone tissue. This oral micro/nano hydrogel microsphere system offered a new approach for preventing postmenopausal osteoporosis. On this basis, our study created an ultrasound‐triggered tissue penetration hydrogel. This study can effectively solve the problem of permeability impedance of damaged tissue faced by the above studies. Therefore, the domestic and foreign research on bone tissue microvascular reconstruction carried out a systematic investigation, and made a series of achievements. We also made a summary in Table S1 (Supporting Information) based on our own research.

In the study, we constructed an ultrasound‐triggered tissue‐permeable hydrogel that can reconstruct microvascular networks in the tibia of osteoporotic rats. In the part of in vitro exploration, we observed that the gene transfection efficiency and in vitro vascularization function of US+PI3K‐Lip group without hydrogel were significantly higher than those of US+Gel‐PI3K‐Lip group with hydrogel sustained‐release function. However, in vivo experiments have shown the opposite result. The study proved that US+Gel‐PI3K‐Lip group had better therapeutic effect in vivo. The principle of the above phenomenon is mainly that the PI3K‐Lip liposome complex of US+PI3K‐Lip group can directly transfect endothelial cells in vitro experiments. Therefore, the transfection efficiency of US+PI3K‐Lip group was higher than US+Gel‐PI3K‐Lip group. However, in vivo experiments, compared with the US+PI3K‐Lip group, the US+Gel‐PI3K‐Lip group can slowly release the PI3K‐Lip complex, thus achieving sustained cell transfection in vivo. Therefore, the long‐term remodeling performance of bone microvessels in US+Gel‐PI3K‐Lip group was better. A similar phenomenon was also found in the previous study of Cai et al.^[^ ^33^ ^]^ Therefore, through these studies, we further demonstrated that ultrasound‐triggered tissue permeability hydrogels can significantly promote the reconstruction of microvascular networks.

Fibrin was used as the basis for the formation of tissue permeable hydrogels. After tissue injury, fibrin acts as a natural scaffold that triggers the clotting process and provides the initial substrate for cell attachment, migration, proliferation, and differentiation.^[^ ^34^ ^]^ Therefore, in our study, a mixture of fibrinogen solution was injected into the injured tissue area so that it fully penetrates the injured tissue and its surrounding microvascular network. ultrasound triggered liposomes to release calcium ions and thrombin. Calcium ion changed the energy coordination of transglutaminase, leading to its conformational change, and its conformational change could open the channel and make it activate. Thrombin catalyzed the hydrolysis of fibrinogen to fibrin monomers. The fibrin monomer was catalytically crosslinked by activated transglutaminase through transamidation reactions, resulting in covalent crosslinking of the lysine and glutamine side chain residues of the fibrin molecules. Ultrasound techniques were used to trigger the intrachistological formation of hydrogels and effectively penetrate the tissues to achieve efficient gene transfection. In recent years, ultrasound and ultrasonic functional biomaterials have provided powerful and convenient technologies for precision medicine, and the research and application have become more and more extensive.^[^ ^22^, ^35^ ^]^ The platform can provide time and space drug release, which plays an extremely important role in the field of drug delivery.^[^ ^36^ ^]^ Therefore, in line with the current research status, we need to use hydrogel as a drug delivery carrier and combine ultrasonic triggering technology to achieve accurate drug delivery, and further solve the existing clinical treatment difficulties of bone microvascular reconstruction.

In our study, a syringe was used to inject a hydrogel precursor into the tibia tissue of osteoporotic rats, after which the hydrogel formation was triggered by ultrasound. In addition, marketed clinical injection therapy devices such as Shusuda and ArrowEZ‐IO can be mixed with a variety of solutions for local injection therapy of bone tissue. Therefore, it is also feasible to achieve local microvascular reconstruction by injecting the ultrasonic response hydrogel into the tibia of rats. In addition, Ma et al.^[^ ^37^ ^]^ By changing the concentration of hydrogel precursors and precisely adjusting the injection performance of the hydrogel system, the solution can be retained, diffused and successfully cross‐linked in the bone trabecular space. Yao et al.^[^ ^28a^ ^]^ injected a precursor solution containing calcium flavin into the tibia of osteoporotic mice for 30 min. They observed permeable green fluorescent signals in the bones of mice with osteoporosis. After 2 h of observation, the green fluorescence signal has fully penetrated the bone tissue. Therefore, these studies demonstrated the effectiveness and feasibility of local bone injection and formation of hydrogels.

Phosphatidylinositol 3‐kinase (PI3K) can regulate the metabolism of various lipids through intracellular signals, and then play a role in cell proliferation, survival, and differentiation. PI3K signaling is essential for cardiovascular homeostasis and angiogenesis. PI3K, as a key center, mediates angiogenesis of AKT transduction, thereby promoting the generation of angiogenic signals. Notably, PI3K can be activated through HIF‐1‐dependent and non‐dependent pathways.^[^ ^38^ ^]^ In addition, HIF‐1 can be expressed under hypoxic environmental conditions, promoting VEGF expression and PI3K signal transduction, and ultimately mediating angiogenesis. In addition, other angiogenic factors can also be overexpressed by PI3K‐mediated, thus promoting angiogenesis.^[^ ^39^ ^]^ Therefore, in our study, we found that overactivation of PI3K can activate AKT and HIF‐1*α* overexpression. At the same time, e‐NOS was also over‐activated, which is consistent with the results of previous studies.

## 4 Conclusion

In summary, our hydrogel precursor solution efficiently penetrated the damaged tissue area, and subsequently ultrasound triggered the release of thrombin from liposomes in the solution to hydrolyze fibrinogen, and triggered the formation of fibrin solid hydrogel network in situ with calcium ions as well as transglutaminase as catalysts. This method effectively addressed the permeation impedance bottleneck of the damaged tissues, and ultimately promoted the formation of the microvascular network in the tissues significantly. Therefore, our ultrasound‐triggered hydrogel can be utilized as a promising tissue permeation biomaterial, offering a novel approach for microvascular network reconstruction.

## 5 Experimental Section

### Materials

2,3‐Dioleoyloxy‐propyl‐trimethylammonium‐chloride (DOTAP, Pharmaceutical Tech, China); Lecithin (Macklin, China); 1, 2‐Dioleoyl‐Sn‐Glycero‐3‐phosphoethanolamine (DOPE, Highfine, China); Fibrinogen (Yeasen,China); Live/Dead Cell Kit (Servicebio, China); Calcium Ion Color Development Kit (Beyotime, China); DAPI ((4',6‐diamidino‐2‐phenylindole) Solarbio, China); Chloroform (Aladdin, China); Calcium chloride (Aladdin, China); mPEG‐DSPE (1,2‐distearoy‐sn‐glycero‐3‐phosphoethanolamine‐N‐[methoxy(polyethylene glycol)]) (shyuanye, China); CCK‐8 Kit (Servicebio, China); RIPA Lysate (Servicebio, China); Cholesterol (Aladdin, China), et al.

### Preparation and Characterization of Liposomes

The process was as follows: First, DSPE‐PEG‐Mal was dissolved in DMF and the 289 W polypeptide (Apeptide, Shanghai, China) was fully dissolved in PBS and stirred at room temperature for 4 h. Next, the mixed solution was put into a 3.5 kDa dialysis bag for full dialysis to remove the unbound 289 W. The purified 289W‐PEG‐DSPE was freeze‐dried by a freeze‐drying machine. After that, 289W‐PEG‐DSPE, DOTAP, DOPE, and cholesterol were fully dissolved in 1 mL chloroform according to molar ratio 2:30:40:30. Then vacuum drying was carried out at 60 °C using a rotary evaporator. The 20 mg mL^−1^ CaCl_2_ solution, thrombin complex, was added into the dry lipid membrane. Next, the mixture was hydrated by 10% energy ultrasonic wave, and then the polydispersed liposome structure was obtained. The isotonic solution was mixed and dialyzed to remove the free calcium. The liposomes not modified by 289 W were used as controls, and other preparation processes were the same as above. Transmission electron microscopy was used to investigate the diameter and morphology of the liposomes. The particle size and Zeta potential of the liposomes were characterized using Zetasizer (Malvern Nano‐ZS, UK) instrument. Before test, the liposomes were diluted and dissolved in isotonic buffer.

### Preparation and Transfection Efficiency Evaluation of Loaded DNA Liposomes

In this study, HUVECs were cultured in 24‐well plates.^[^ ^40^ ^]^ The cell fusion rate was 70% before further experiment. The proportions of plasmids: liposomes were 1:1, 5:1, 10:1, 20:1 in sequence. In this part of the experiment, the control group was plasmid free group. After the mixture of the above liposomes and plasmids, the cells were incubated with serum‐free medium of 50 µm for 30 min, and then the cells were incubated with serum‐free medium for 6 h. Finally, Lip‐PI3K‐289w liposomes and plasmids were co‐incubated with cells by adding serum medium for 48 h. Lip‐pi3k‐289 W was Lip‐PI3K. After 48 h, the cells were washed three times with PBS. The nucleus was stained using DAPI according to the instructions. After 30 min, the HUVECs were washed with PBS for three times. Fluorescence analyzed the transfection efficiency. The fluorescence colocalization repetition rate of different groups was quantitatively analyzed by ImageJ 1.8.0 software, and transfection rate was evaluated after statistical analysis.

### Western Blot was used to Detect the Proteins Expression

Western blotting showed that PI3K gene was successfully transfected and expressed. First, the BCA protein kit (Beyotime, P0012, China) was used to detect the concentration of total protein. Next, the treated sample protein samples were sampled and electrophoreted and transferred to (Millipore, 0.45 µm, USA) membranes. Polyvinylidene fluoride (PVDF) membrane was treated with protein sealing solution for 1 h to prevent non‐specific binding of proteins. The primary antibody of PI3K was Abcam and the dilution was 1:1000. Then the PVDF membrane was washed with Tris Buffered Saline with Tween (TBST) for three times to remove the free primary antibody. The corresponding secondary antibody (1:10,000) was incubated for 1 h, and then TBST was used to wash the free secondary antibody. Finally, the luminescence system was used for detection and imaging. Statistical analysis was performed using ImageJ software and graphpad prism.^[^ ^41^ ^]^

### Ultrasound Triggers the Release of Ions and the Formation of Tissue Permeable Hydrogels

The use condition of ultrasonic therapy instrument was frequency 1MHZ, intensity 2 W. The duration of the ultrasonic probe was 1000 s, and the calcium concentration was determined with the calcium ion color developing kit (S1063S, Beyotime), and the release curve was plotted (S1063S, Beyotime). First, 0.1 mm fibrinogen and 8.69 mm transglutaminase were dissolved in 0.9% NaCl. Second, the 100 µL complex liposome prepared in the above part was mixed into the solution system to prepare the fibrinogen complex solution. Third, thrombin was released from liposomes after ultrasound triggering, activating a series of gelation pathways. Finally, the time and state of hydrogel formation were observed, recorded, and photographed. If the liposome did not carry the relevant gene, it was a US+Gel‐Lip. Without ultrasonic action, each component was directly mixed to form hydrogel, which was Gel group.

### Characterization of Ultrasound‐Triggered Tissue Permeable Hydrogels

The modulus of the hydrogel was measured at 37 °C by the HAAKE MARS III (USA) rheometer. FTIR and scanning electron microscopy (SEM) were used to investigate the chemical changes, surface morphology and liposome structure of hydrogels. The mechanical properties of the composite hydrogel were tested at the size of (7 mm × 7 mm × 10 mm) by the universal measuring instrument Instron5967 (Instron, Norwood, MA). The test parameters were: 10 N capacity sensor, 60 ± 5% humidity and 25 ± 0.5 °C. The instrument was loaded until the hydrogel breaks, and the maximum stress load of the hydrogel was finally measured.

### Biocompatibility Evaluation of Ultrasound‐Triggered Tissue Permeable Hydrogels

In the control group, PBS buffer was added into the cell medium. US+Gels‐PI3K‐Lip group was genetically engineered hydrogel. US+PI3K‐Lip plasmid‐liposome complex can directly transfect cells. US+Gels‐Lip was a complex hydrogel that did not contain genes. Ultrasound was used at a condition of 100 s, an intensity of 1 W, and a frequency of 1 MHz to trigger each set of samples. According to the instructions, the activity of HUVECs was detected by a live and dead staining kit. The cell status was recorded by fluorescence microscope (Nikon, Japan), and the relevant statistical analysis was performed.

### Cell Migration Performance In Vitro

1 × 10^5^ HUVECs were inoculated into 24‐well plates, and then the extracts of each group of samples were co‐cultured with cells. The 200 µL sterile pipette tip was used to scratch the cells to form a line wound. After incubation for 0, 24 and 48 h, the cell migration was observed by fluorescence microscopy. Image J software was used for quantitative analysis. In addition, 600 µL of 20% serum cell medium was added to the middle and lower chamber of the Transwell plate. The cells in the upper chamber were re‐suspended and 200 µL cell suspension was added. After 24 h, the cells were fixed and stained with 10% crystal violet. Finally, the number of cells was calculated and averaged.

### Tubular Properties Evaluation of Ultrasound Triggered Tissue Penetration Hydrogel

The 24‐well confocal plate was coated with Matrigel (Corning Inc., NY). After 30 min of constant temperature fixation, HUVECs were inoculated on matrix glue and then added to DMEM medium for incubation for 1, 3, and 6 h. The formation of tubes was observed by fluorescence microscope, and the quantitative analysis was carried out by Image J software.

### Evaluation of In Vivo Ultrasound‐Triggered Tissue Permeable Hydrogels for Promoting Microvascular Network Regeneration

In the previous article, the establishment of an animal model for osteoporosis^[^ ^42^ ^]^ was discussed. In a rat model of osteoporosis, microvessels in the cancellous bone region of the femur were significantly reduced. To evaluate the ability of biomaterials to reconstruct bone microvessels, SD rats were anesthetized with isoflurane combined with a gas anesthesia machine.^[^ ^43^ ^]^ In the preceding study, a syringe was employed to penetrate the metaphyseal tibia bone region, allowing for fluid injection in each group. The results showed that ultrasound triggered the formation of hydrogel in cancellous bone region. In recent years, many studies have shown that local treatment can be effectively achieved by minimally invasive injection of various liquid biological materials into the tibia.^[^ ^28^ ^]^ In addition, domestic and foreign clinical therapeutic instruments such as ArrowEZ‐IO and Shusuda have been used for local injection of many mixed solutions. Therefore, it was possible to achieve local vascular reconstruction by injecting ultrasound‐responsive materials into the tibia of rats. In addition, an in vitro experiment was designed to explore the tissue permeability of the fibrinogen complex solution. The composite solution was added to the artificial bone and the permeability of the mixture was observed. After the mixed solution was successfully cross‐linked in artificial bone, it was placed in a liquid environment to observe the stability of the hydrogel. Our Animal Ethics Approval number is: SHDSYY‐2020‐4297.

### Histological and Immunofluorescence Analysis

Rat tibia specimens were immersed in a 4% polyformaldehyde solution for 48 h, then moved to an Ethylenediaminetetraacetic acid (EDTA) solution for decalcification. After decalcification was complete, dehydration and embedding were carried out. 5 µm thickness slices were performed after embedding. Animal sections were stained with H&E and immunofluorescence, and the corresponding antibodies were PI3K, Akt, HIF‐1*α* and e‐NOS (Servicebio, China). Then, the secondary antibody of the corresponding species (Servicebio, China) was processed for 1 h, and the images were observed by fluorescence microscope. Finally, Image J was used for quantitative processing.

## Conflict of Interest

The authors declare no conflict of interest.

## Supporting information

Supporting Information

## Data Availability

The data that support the findings of this study are available from the corresponding author upon reasonable request.
