# Supplementary material for: Tissue‐Penetrating Ultrasound‐Triggered Hydrogel for Promoting Microvascular Network Reconstruction
Source: Adv Sci (Weinh). 2024 Apr 10;11(23):2401368. doi: 10.1002/advs.202401368 (PMC11187930; doi:10.1002/advs.202401368)
Supplement: Supplementary file 1 — Supporting Information [file ADVS-11-2401368-s001.docx]

DOI: 10.1002/ ((No. advs.202401368))

**Article type: Article**

**Tissue-penetrating ultrasound-triggered hydrogel**

**for promoting microvascular network reconstruction**

*Zhenyu Zhao*^1 †^*, Yin Zhang*^2 †^*, Chen Meng*^2 †^*, Xiaoyun Xie*^1^**, Wenguo Cui*^2^**, Keqiang Zuo*^1^***

^1^ Department of Interventional and Vascular Surgery, Shanghai Tenth People's Hospital, Tongji University School of Medicine, Shanghai 200072, China.

^2^ Department of Orthopaedics, Shanghai Key Laboratory for Prevention and Treatment of Bone and Joint Diseases, Shanghai Institute of Traumatology and Orthopaedics, Ruijin Hospital, Shanghai Jiao Tong University School of Medicine, 197 Ruijin 2nd Road, Shanghai 200025, China.

^⁎^Corresponding authors.

E-mail addresses: xiaoyunxietj@126.com (X. Xie); 1805100@tongji.edu.cn (K. Zuo); wgcui@sjtu.edu.cn (W. Cui)

^†^These authors contributed equally to this work.


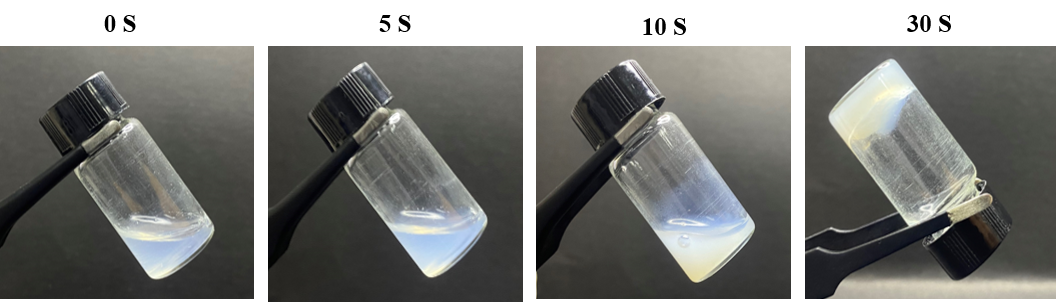


Figure. S1. The formation time of hydrogel was observed after ultrasonic triggering.


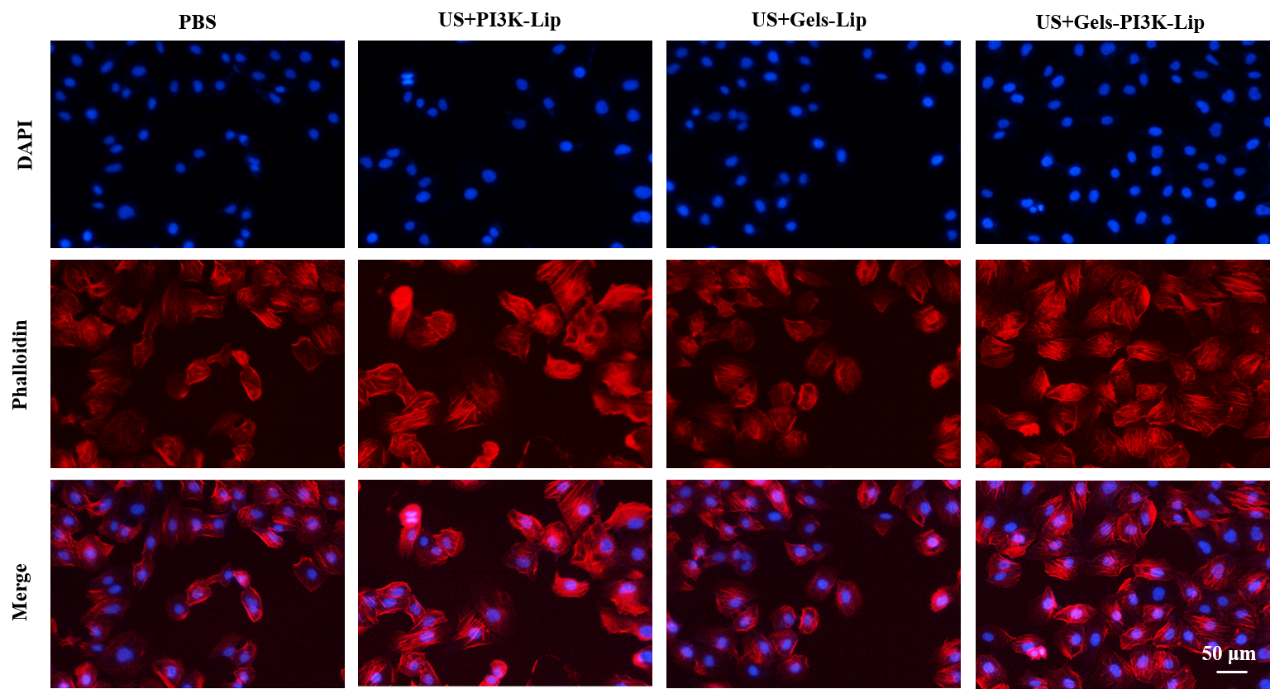


Figure. S2. Changes of cell morphology after co-incubation of samples and cells in each group.


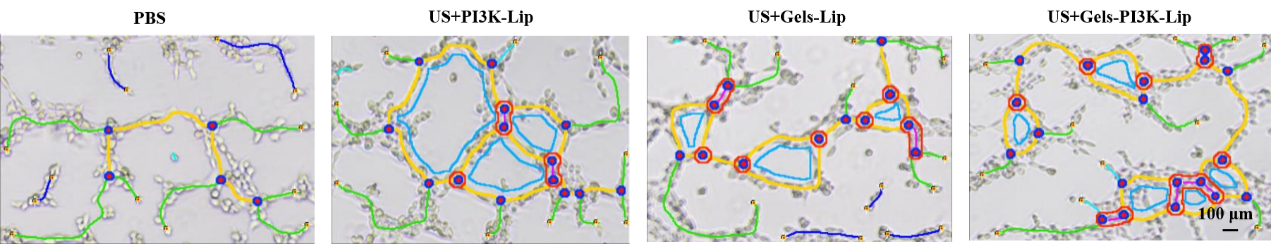


Figure. S3. The angiogenic ability of endothelial cells was analyzed statistically.


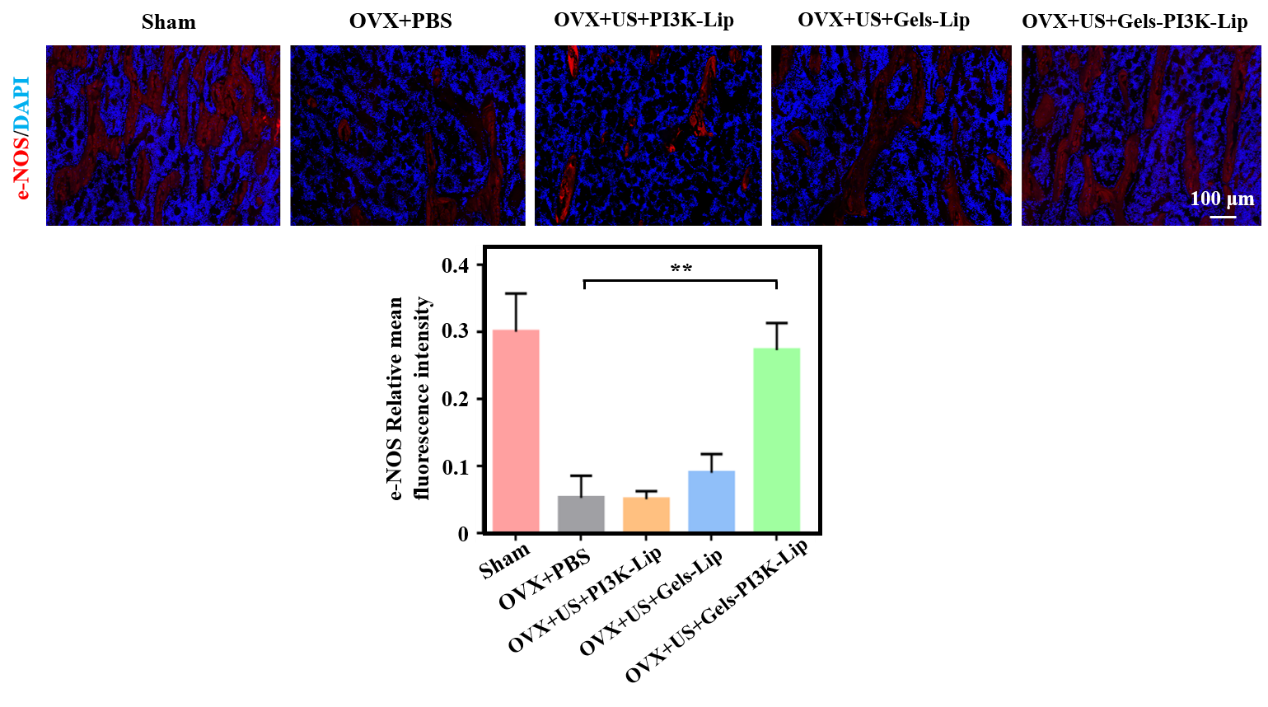


Figure. S4. The expression of e-NOS was detected by immunofluorescence staining, and statistical analysis was performed.

Table. S1. Our study was compared with the results of other relevant studies.

| The main material of hydrogel | Hydrogel crosslinking mechanism | Techniques for triggering hydrogel crosslinking | Delivery methods of biological materials | Mechanism of bone angiogenesis | Innovation | Ref. |
| --- | --- | --- | --- | --- | --- | --- |
| Type I collagen and fibronectin | Protein folding and assembly | Spontaneous crosslinking | Injection | Desferriamine promotes H-type angiogenesis | The local application of deferriamine was promoted. This study provides a theoretical basis for the treatment of large segmental defect of femur by targeting H-type vessels. | [1] |
| Chondroitin sulfate methacrylate | The amino group forms a covalent bond with the carboxyl group | Crosslinking is triggered at 60 °C | No in vivo experiments were involved | No in-depth exploration | Covalent cross-linked organic-inorganic composite hydrogel with strong mechanical properties promotes bone microvascular regeneration | [2] |
| Extracellular matrix | Not mentioned | Spontaneous crosslinking | Injection | Macrophage chemotaxis and M2 polarization promote bone reconstruction | Early immune regulation of biomaterials enhances angiogenesis and osteogenesis | [3] |
| Sodium alginate | Electrostatic interaction | Spontaneous crosslinking | Oral administration | Type H vessel formation and inhibit bone resorption, effectively mitigating bone loss by activating HIF-1𝜶/VEGF signaling pathway and promoting heme oxygenase-1 expression | Oral microspheres promote osteoporotic bone reconstruction by regulating H-type blood vessels | [4] |
| Gelatin methacryloyl and poly (ethylene oxide) | Covalent crosslinking | Ultraviolet | Implantation | Osteogenic components Decalcified bone matrix granules and VEGF promote osteogenesis/angiogenesis | Bone biomimetic porous hydrogel scaffolds provide a new strategy for ectopic vascularization tissue engineering. | [5] |
| Amyloid fibrils and clay nanosheets with DNA strands | Electrostatic and hydrogen bonding | Self-assembly | Implantation | The peptides stimulate the tubular formation and migration of HUEVCs. Si^4+^ and Mg^2+^ promoted bone formation. | Amyloid fibers and clay nanosheets are co-assembled with supramolecules of DNA strands to construct hydrogels | [6] |
| Vascular derived extracellular matrix | Covalent crosslinking | Ultraviolet | Implantation | BMP-2 release and angiogenic properties of extracellular matrix | Vasogenic extracellular matrix synergistically enhances BMP-2 therapeutic index | [7] |
| Fibrinogen | Covalent crosslinking | Ultrasound | Injection | PI3K gene engineering therapy | Tissue permeability ultrasound triggered hydrogel, which effectively solved the problem of damaged tissue permeability impedance faced by the above studies. | Our research |

[1] Y. W. Zeng, C. Huang, D. M. Duan, A. J. Lou, Y. Guo, T. H. Xiao, J. G. Wei, S. Liu, Z. Wang, Q. H. Yang, L. Zhou, Z. H. Wu, L. Wang, *Acta Biomater.* **2022**, *153*, 108, <https://doi.org/10.1016/j.actbio.2022.09.018>.

[2] F. M. Zhang, L. Zhou, Z. N. Zhou, C. Dai, L. Fan, C. H. Li, C. R. Xiao, C. Y. Ning, Y. Liu, J. Q. Du, G. X. Tan, *Biopolymers* **2019**, *110* (12), <https://doi.org/10.1002/bip.23328>.

[3] P. C. Qiu, M. B. Li, K. Chen, B. Fang, P. F. Chen, Z. B. Tang, X. F. Lin, S. W. Fan, *Biomaterials* **2020**, *227*, <https://doi.org/10.1016/j.biomaterials.2019.119552>.

[4] J. J. Li, G. Wei, G. W. Liu, Y. W. Du, R. Z. Zhang, A. F. Wang, B. S. Liu, W. G. Cui, P. Jia, Y. J. Xu, *Adv. Sci.* **2023**, *10* (15), <https://doi.org/10.1002/advs.202207381>.

[5] X. D. Wu, Y. Y. Huo, Z. Ci, Y. H. Wang, W. Xu, B. S. Bai, J. X. Hao, G. H. Hu, M. Y. Yu, W. J. Ren, Y. X. Zhang, Y. J. Hua, G. D. Zhou, *Appl. Mater. Today* **2022**, *27*, <https://doi.org/10.1016/j.apmt.2022.101478>.

[6] Q. Yang, Y. L. Miao, J. S. Luo, Y. H. Chen, Y. J. Wang, *ACS nano* **2023**, *17* (17), 17131, <https://doi.org/10.1021/acsnano.3c04816>.

[7] J. Y. Chen, X. Z. Zhou, W. Q. Sun, Z. J. Zhang, W. S. Teng, F. Q. Wang, H. X. Sun, W. Zhang, J. W. Wang, X. H. Yu, Z. M. Ye, W. X. Li, *"Small (Weinheim an der Bergstrasse, Germany)"* **2022**, *18* (36), <https://doi.org/10.1002/smll.202107991>.
